# SMS text pre-notification and delivery of reminder e-mails to increase response rates to postal questionnaires in the SUSPEND trial: a factorial design, randomised controlled trial

**DOI:** 10.1186/s13063-015-0808-9

**Published:** 2015-07-08

**Authors:** Kathryn Starr, Gladys McPherson, Mark Forrest, Seonaidh C. Cotton

**Affiliations:** Centre for Healthcare Randomised Trials, Health Services Research Unit, University of Aberdeen, Health Sciences Building, Foresterhill, Aberdeen, AB25 2ZD Scotland

**Keywords:** Questionnaires, Non-response, Randomised controlled trial, Short messenger service (SMS), Electronic mail (e-mail)

## Abstract

**Background:**

Patient-reported outcomes are vital in informing randomised controlled trials (RCTs) and health-care interventions and policies from the patient’s perspective. However, participant non-response may introduce bias and can affect the generalisability of the trial.

This study evaluates two interventions aimed at increasing response rates to postal questionnaires within a large, UK-wide RCT: pre-notification via short messenger service (SMS) text prior to sending the initial mailing of trial questionnaires versus no pre-notification; for non-responders to the initial mailing of the questionnaires, an e-mail reminder (containing a hyperlink to complete the questionnaire online) versus a postal reminder.

**Methods:**

This study is a 2×2 partial factorial design RCT nested within an RCT of medical expulsive therapy for ureteric stone disease. Participants who supplied a mobile telephone number were randomly assigned to receive an SMS text pre-notification of questionnaire delivery or no pre-notification. Those who supplied an e-mail address were randomly assigned to receive a questionnaire reminder by e-mail or post. Participants could be randomly assigned to the pre-notification comparison or the reminder comparison or both. The primary outcome measure was response rate at each questionnaire time point.

**Results:**

Four hundred eighteen participants were randomly assigned to the SMS pre-notification comparison (80 % were male, and the mean age was 41 years with a standard deviation (SD) of 11.1). The intervention had no effect on response rate at either questionnaire time point. In subgroup analyses, SMS pre-notification increased response rates in women but only at the first questionnaire time point. One hundred nineteen participants were randomly assigned to the reminder comparison (80 % were male, and the mean age was 42 years with an SD of 12.1). There was no difference in response rate in those who received an e-mail reminder compared with those who received a postal reminder.

**Conclusions:**

SMS text pre-notification of questionnaire delivery and email delivery of questionnaire reminders did not improve response rates. There was some evidence to suggest that SMS text pre-notification may be effective in women, and further studies to investigate this may be warranted. E-mail reminders for participants to return their postal questionnaire could be advantageous given that response rates were similar following either type of reminder and the low cost of delivering an e-mail compared with a postal reminder.

This is a substudy of the SUSPEND trial (ISCTRN69423238) (18 Nov. 2010).

**Electronic supplementary material:**

The online version of this article (doi:10.1186/s13063-015-0808-9) contains supplementary material, which is available to authorized users.

## Background

Patient perspectives should provide evidence to inform the design, delivery, and evaluation of health care [1]. The knowledge that can be gained from informing health outcomes with evidence from the patient’s perspective means that patient-reported outcomes are key in many randomised controlled trials (RCTs) of health-care interventions. However, these are often collected by postal questionnaires, and participant non-response can reduce effective sample size, potentially introduce bias, and impact on the generalisability of the trial results [2]. There is, therefore, a need for researchers to encourage participants to respond to questionnaires and deliver sound, unbiased trials in a timely and resource-efficient manner to provide evidence that informs health-care policies and guidelines and, ultimately, better public health.

Many ways have been suggested to improve response rates to questionnaires, and these are discussed in two Cochrane reviews [3, 4]. Both reviews include RCTs of strategies to improve questionnaire response rates; however, the more recent review [4] is limited to those conducted within parent RCTs. The overwhelming evidence in both of these reviews, and in another review within population-based cohort studies [5], supports the use of monetary incentives in improving response rates. However, the use of monetary incentives carries practical and ethical considerations, especially within studies that are publically funded.

In 2012, surveys by the Office of National Statistics reported that 91 % of the UK population owned a mobile phone and that 16- to 24-year-olds were most likely to possess one [6]. E-mail use is also widespread: 75 % of the UK population in 2013 used the internet to send and receive e-mails [7]. The use of e-mail was most common amongst 25- to 34-year-olds (of whom 89 % accessed the internet for e-mail), and men were more likely to use e-mail than women (78 % versus 72 %). These data are of particular interest given that younger people are less likely to respond to questionnaires than older people [8, 9] but are also more likely to use mobile phones and e-mail. However, there is a lack of evidence about the use of mobile telephone and e-mail technology to influence response rates in clinical trials. The purpose of the study described here was to investigate whether technology favoured by younger age groups could be used to increase response rates to questionnaires in the SUSPEND trial, a large RCT of medical expulsive therapy for ureteric stone disease [10]. The hypothesis for the SUSPEND response rate study (SUSRes) is that the use of SMS text pre-notification of questionnaire delivery and e-mail delivery of questionnaire reminders (with a link to complete the questionnaire in a secure web site) will improve questionnaire response rates.

## Methods

SUSRes was a randomised, controlled, 2×2 partial factorial design nested within the SUSPEND RCT [10], testing two methods—the use of SMS text pre-notification of questionnaire delivery and e-mail delivery of questionnaire reminders (with a link to complete the questionnaire in a secure web site)—to improve questionnaire response rates. Within SUSPEND, patient-reported outcomes were collected by postal questionnaire at 4 and 12 weeks, and SUSRes was conducted in 24 SUSPEND sites throughout the UK from June 2012 to March 2014. SUSRes was not planned at the outset of the SUSPEND RCT; rather, it was developed in response to lower-than-expected response rates to postal questionnaires within SUSPEND.

### Ethics and other approvals

The study was reviewed and approved by the East of Scotland Research Ethics Service as a substantial amendment to the SUSPEND trial (Research Ethics Committee) reference 10/S0501/31) and received approval from local research and development departments at the SUSPEND sites (Aberdeen Royal Infirmary; Addenbrooke’s Hospital, Cambridge; Bristol Royal Infirmary; Broadgreen Hospital, Liverpool; Cheltenham General Hospital; Derriford Hospital, Plymouth; Freeman Hospital, Newcastle upon Tyne; Guy’s Hospital, London; Manchester Royal Infirmary; Morriston Hospital, Swansea; Norfolk and Norwich University Hospital; Pinderfields Hospital, Wakefield; Queen Elizabeth Hospital, Birmingham; Raigmore Hospital, Inverness; Royal Hallamshire Hospital, Sheffield; Southampton General Hospital; Southmead Hospital, Bristol; St George’s Hospital, London; St James’s University Hospital, Leeds; Sunderland Royal Hospital; The James Cook University Hospital, Middlesbrough; Torbay Hospital, Torquay; University Hospital of South Manchester; Western General Hospital, Edinburgh) before commencing. Participants provided written informed consent for SUSPEND which encompassed the SUSRes study.

### Participants

Participants who were newly randomly assigned to the SUSPEND trial, had not reached the 4-week time point, and were willing to supply a mobile phone number or an e-mail address (or both) were considered for the study. Participants could be included in one or both comparisons. All participants who met the inclusion criteria and where a mobile phone number was recorded were included in the SMS text comparison. Participants for whom an e-mail address was provided were included in the e-mail comparison. All participants provided written informed consent for the SUSPEND trial and therefore this study.

### Interventions and controls

There were two comparisons (each with a separate randomisation) within the SUSRes study.

#### SMS text pre-notification comparison

All participants randomly assigned to the intervention arm were sent an SMS text message pre-notification of the delivery of the initial 4- and 12-week questionnaires. The message was generated automatically from the SUSPEND trial database and sent via an external supplier the same day the questionnaire was dispatched from the trial office. The SMS read: “Many thanks for participating in the SUSPEND trial. You will shortly receive your xx-week questionnaire. We hope you can take a few minutes to complete this and return it to us”. Participants randomly assigned to the control arm were not sent any pre-notification of the delivery of the 4- and 12-week questionnaires.

#### E-mail reminder comparison

All participants who were randomly assigned to the intervention arm and who did not respond to the initial 4- or 12-week questionnaire received an e-mail which included a link to complete the questionnaire online or were invited to return the paper copy if they wished. Participants who were randomly assigned to the control arm and who did not respond to the initial 4- or 12-week questionnaire received their reminder by post with a further copy of the questionnaire.

Both types of reminder would have been generated on the same day, two weeks after the initial questionnaire was sent out by post from the trial office, by the SUSPEND trial database. The e-mails were automatically sent from the trial database and the postal reminders were printed and posted by trial office staff on the same day.

The wording of the reminder e-mail reflected the wording and layout of the letter that accompanied the postal questionnaires to avoid any bias by modifying this. The online questionnaire contained the same questions as the postal questionnaire.

### Allocation and randomisation

Participants were randomly allocated to the intervention or control groups of the SUSRes study on a 1:1 basis by using a computer-generated system that was concealed and remote from the users. The randomisation algorithm was permuted blocks (block size of 4) stratified by age (≤ 40 years or > 40 years) and sex as these variables are known to affect response rate [8, 9]. The allocated group applied to both SUSPEND questionnaire time points (4 and 12 weeks). Owing to the nature of the intervention, it was not possible to blind the participants or trial office staff to allocation; however, the researchers remained blind.

### Outcomes

The primary outcome for both comparisons was defined as questionnaire response rate at each time point. There were no secondary outcomes.

### Sample size

The sample size for the SUSRes study was dictated by the number of participants still to be recruited into SUSPEND at the time the study started (710); therefore, no formal sample size calculation was undertaken.

### Statistical analysis

All analyses were performed in Microsoft Excel (2010; Microsoft Corporation, Redmond, WA, USA) and IBM SPSS statistics version 22.0 (IBM Corporation, Somers, NY, USA). Two comparisons were made: SMS text message pre-notification versus no pre-notification of questionnaire delivery and questionnaire e-mail reminder versus postal reminder.

Participant baseline characteristics (age and sex) between the intervention and control groups within each intervention comparison were compared by using an independent *t* test or chi-squared test as appropriate.

The primary outcomes (response rate at each time point) were analysed on the basis of the intention-to-treat principle [11], and all participants were analysed as randomly assigned. To assess the impact of each intervention, adjusted odds ratios (ORs) (adjusted for the stratification variables age and sex) with 95 % confidence intervals (CIs) were computed by using logistic regression. Allocation to the other comparison was also added as a co-variate in the adjusted analysis. Possible interaction between the two interventions was explored with logistic regression analysis in those participants who had been randomly assigned in both comparisons.

The effects of age and sex were considered in pre-specified subgroup analyses by using the same analysis techniques described above and adjusted for age or sex as appropriate and allocation to the other comparison.

The CONSORT (Consolidated Standards Of Reporting Trials) checklist [12] for this study can be found in Additional file 1.

## Results

**Fig. 1:**
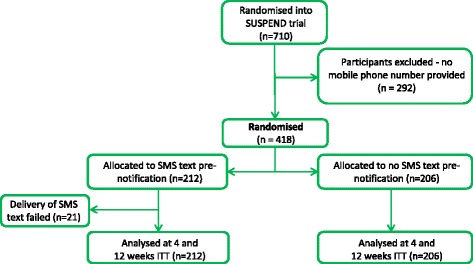
CONSORT (Consolidated Standards Of Reporting Trials) diagram: pre-notification comparison. ITT = Intention-to-treat

**Fig. 2:**
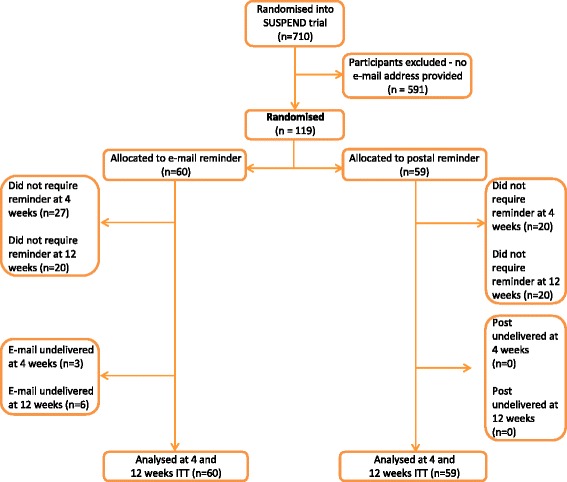
CONSORT (Consolidated Standards Of Reporting Trials) diagram: reminder comparison. ITT = Intention-to-treat

The flow of participants through the SUSRes study is shown in Figs. 1 and 2 according to the recommendations of the CONSORT statement [12]. In total, 710 participants who entered the SUSPEND RCT were assessed for eligibility to enter the SUSRes study. Of those, 418 (59 %) were eligible to be randomly assigned for the pre-notification comparison and 119 (17 %) were eligible to be randomly assigned to the reminder comparison. Within the pre-notification comparison, 80 % were male and the mean age was 41 years (standard deviation (SD) of 11.1). Within the reminder comparison, 80 % were male and the mean age was 42 years (SD of 12.1). Within each comparison, the randomised groups were well balanced (Table 1) and there were no statistically significant differences between the intervention and control groups.

**Table 1:** SUSRes study participant baseline characteristics

	SMS text pre-notification comparison		E-mail reminder comparison	
	SMS text pre-notification *n* = 212	No pre-notification *n* = 206	E-mail reminder *n* = 60	Postal reminder *n* = 59
Age				
Mean, years	40.7	40.8	42.9	42.1
SD	11.06	11.25	12.78	11.40
Median age, years	41	41	45	45
Age range, years	20–65	18–65	21–65	19–65
Age categories				
≤40 years, n	98	100	34	34
%	46 %	49 %	57 %	58 %
>40 years, n	114	106	26	25
%	54 %	51 %	43 %	42 %
Sex				
Male, n	171	164	48	46
%	81 %	80 %	80 %	78 %
Female, n	41	42	12	13
%	19 %	20 %	20 %	22 %

*SMS* short messenger service, *SD* standard deviation

At 4 weeks, the questionnaire response rate was slightly higher (57 %) following an SMS text pre-notification than in the control group (no SMS text: 52 %), but the difference was not statistically significant (adjusted OR 1.24, 95 % CI 0.84–1.82; Table 2). There was no effect of an e-mail reminder compared with a postal reminder on questionnaire response rates (68 % versus 66 %, respectively) at 4 weeks (adjusted OR 1.11, 95 % CI 0.51–2.40; Table 2). There was no effect of either intervention at 12 weeks (Table 2).

**Table 2:** Questionnaire response rate within treatment groups by questionnaire time point

Questionnaire time point	SMS text pre-notification *n* = 212	No pre-notification *n* = 206	E-mail reminder *n* = 60	Postal reminder *n* = 59
Four weeks: n/N	121/212	106/206	41/60	39/59
%	57 %	52 %	68 %	66 %
Adjusted odds ratio (95 % CI)	1.24 (0.84–1.82)^a^; *P* = 0.290		1.11 (0.51–2.40)^b^; *P* = 0.796	
Twelve weeks: n/N	89/212	87/206	33/60	32/59
%	42 %	42 %	55 %	54 %
Adjusted odds ratio (95 % CI)	0.97 (0.66–1.44)^a^; *P* = 0.895		1.03 (0.49–2.15)^b^; *P* = 0.937	

Data are presented as number and percentages within each subcategory

*SMS* short messenger service, *CI* confidence interval

^a^Adjusted for age, sex, and reminder allocation

^b^Adjusted for age, sex, and pre-notification allocation

Subgroup analysis of response by sex and age suggested that women were more likely to respond to the 4-week questionnaire following an SMS text pre-notification (adjusted OR 2.58, 95 % CI 1.05–6.33; Table 3). There was no effect of an e-mail reminder in sex or age subgroup analysis (data not shown).

**Table 3:** Questionnaire response rate by sex and age following intervention

	Four-week questionnaire		12-week questionnaire	
	SMS text pre-notification	No pre-notification	SMS text pre-notification	No pre-notification
Male: n/N	90/186	96/186	72/141	69/141
%	56 %	55 %	40 %	44 %
Adjusted^a^ odds ratio (95 % CI)	1.05 (0.68–1.61); *P* = 0.842		0.85 (0.55–1.32); *P* = 0.475	
Female: n/N	16/41	25/41	15/35	20/35
%	61 %	38 %	49 %	36 %
Adjusted^a^ odds ratio (95 % CI)	2.58 (1.05–6.33); *P* = 0.038		1.72 (0.71–4.17); *P* = 0.232	
≤40 years: n/N	43/96	53/96	33/69	36/69
%	54 %	43 %	37 %	33 %
Adjusted^b^ odds ratio (95 % CI)	1.47 (0.83–2.56); *P* = 0.184		1.14 (0.63–2.05); *P* = 0.664	
>40 years: n/N	63/131	68/131	54/107	53/107
%	60 %	59 %	47 %	51 %
Adjusted^b^ odds ratio (95 % CI)	1.04 (0.61–1.79); *P* = 0.877		0.86 (0.51–1.46); *P* = 0.580	

Data are presented as number and percentages within each subcategory

*SMS* short messenger service, *CI* confidence interval

^a^Adjusted for age and reminder allocation

^b^Adjusted for sex and reminder allocation

The factorial trial design allows for an interaction between the two interventions to be evaluated. There was no evidence to suggest an interaction, although the SUSRes study was not powered to detect this.

## Discussion

The aim of the SUSRes study was to provide evidence in relation to the use of SMS text pre-notification of questionnaire delivery and e-mail reminders following questionnaire non-return on response rates within the SUSPEND trial. The SUSPEND trial lent itself to the SUSRes study as the response rate to the trial questionnaires, which collected patient-reported outcomes, was low at the time of the inception of the SUSRes study. Being relatively young [8] and predominantly male [9], which are also the groups more likely to own a mobile phone [6] and use e-mail [7], the trial population was regarded as being one less likely to respond to questionnaires.

SMS text pre-notification failed to have any statistically significant effect on the response rates in the SUSRes trial. This supports previously published data [13, 14], although these studies used different delivery methods for the intervention and different time points of intervention delivery. The observed effect of an SMS text pre-notification in women at 4 weeks may be a chance finding. A bespoke trial database, designed and supported by a dedicated programming team with experience and familiarity with the necessary technology, meant that SMS text pre-notification was relatively straightforward and inexpensive to implement within SUSPEND; however, this may not be as simple to implement in other RCTs. When such technology is available, the small potential benefit of using SMS text pre-notification in women may outweigh the cost of such a strategy: this is worthy of further investigation.

We hypothesised that the use of e-mail reminders, which included a link to complete the questionnaire online, would increase questionnaire response rates. However, this was not the case and the use of e-mail reminders did not have any effect on response rates at 4 and 12 weeks in the SUSRes study; this held true when a post-hoc per-protocol analysis was performed on the participants who required a reminder (e-mail versus postal reminder response rates were 47 % versus 54 % at 4 weeks and 52 % versus 49 % at 12 weeks). This is in contrast to the literature which suggests that, in surveys, e-mail delivery of questionnaires reduces response rate [15]. Given that there was no negative effect of providing questionnaire reminders by e-mail compared with post and that the cost of an e-mail reminder is insignificant (if a trial has a database or information technology system that can manage this) compared with the cost of a postal reminder, this may be a strategy that trialists wish to consider implementing.

Factorial design trials have been used previously in RCTs to investigate methods to improve response rates [16–19]. The SUSRes study differed from a conventional 2×2 factorial design [20] in that, instead of being randomly assigned once to one of four study groups (that is, no pre-notification/postal reminder, no pre-notification/e-mail reminder, pre-notification/postal reminder, or pre-notification/e-mail reminder), participants were subjected to two separate randomisations: no pre-notification versus SMS text pre-notification and postal reminder versus e-mail reminder. The study was conducted in this manner (a partial factorial design) as it was anticipated that participants may not provide both a mobile phone number and an e-mail address and therefore performing two separate randomisations would most efficiently use the available patient population. These fears were well founded as only 56 of the 710 SUSPEND participants (8 %) provided both a mobile phone number and an e-mail address, which would have markedly reduced the sample size.

The main weaknesses of the SUSRes study relate to the number of participants. Firstly, the number who were potentially eligible when the SUSRes study started was limited by the number of participants who had yet to be recruited into the SUSPEND trial (*n* = 710). Because this number was fixed, we did not undertake a formal sample size calculation, and this is a further limitation of our study. Secondly, there was a low recruitment rate to the SUSRes study. Only 59 % (418/710) of those recruited into the SUSPEND trial were eligible for the pre-notification comparison study, and only 17 % (119/710) for the reminder comparison study. There are a number of possible explanations for the low recruitment rate. The patient population in the SUSPEND trial has ureteric stone disease. These patients were recruited to SUSPEND in an acute setting and were often discharged quickly. It is therefore possible that collection of essential trial data (e.g., baseline clinical characteristics) took priority over the collection of information such as e-mail addresses and mobile phone numbers, which may have appeared superfluous to the main trial but were essential for inclusion in the SUSRes study. Anecdotally, some research staff saw themselves as gate keepers of participants’ personal information and were not comfortable collecting these data. Seeking this information directly from the participants may be a solution, particularly if they are also asked about preferred methods of contact. The risk of a type II error (i.e., the risk of failing to detect a difference between two groups when one truly exists) is increased when studies are underpowered, which the SUSRes study is likely to be.

Despite the low recruitment rate to both SUSRes studies, the gender and age (≤ 40 years and > 40 years) of those included in the postal versus e-mail reminder comparison and the gender of those included in the SMS text pre-notification were similar to those of patients who were not included. Potentially reflecting levels of mobile phone ownership, 70 % of those younger than 40 were included in the SMS text pre-notification comparison compared with 53 % of those who were older than 40 years.

Whereas we know how many SUSRes study text messages and e-mail reminders were undelivered and how many postal questionnaires were returned unopened, we do not know how many others were received but not opened or read by the participant. Mobile phone numbers can be shared between people and text messages may not have been received if the mobile phone was switched off or out of service. E-mail messages may have been routinely filtered into junk or spam folders. Postal reminders may have been undelivered (but not returned to the trial office) or discarded.

## Conclusions

SMS text pre-notification of questionnaire delivery and e-mail delivery of questionnaire reminders did not increase questionnaire response rate in the SUSPEND trial population. However, there was some evidence to suggest that SMS text pre-notification may be effective in women, and further studies to investigate this may be warranted. E-mail reminders for participants to return their postal questionnaire could be advantageous given that response rates were similar following either type of reminder, together with the low cost of delivering an e-mail compared with a postal reminder.

## Additional file

Additional file 1:
**CONSORT (Consolidated Standards Of Reporting Trials) 2010 checklist of information to include when reporting a randomised trial.**

## Abbreviations

CI: Confidence interval
CONSORT: Consolidated Standards Of Reporting Trials
OR: Odds ratio
RCT: Randomised controlled trial
SD: Standard deviation
SMS: Short message service
SUSPEND: Spontaneous Ureteric Stone Passage ENabled by Drugs
SUSRes: SUSPEND response rate study
